# Diagnostic Strategy in Older Patients With Unexplained Syncope: Limitations of Preference‐Based Evidence

**DOI:** 10.1111/anec.70199

**Published:** 2026-05-11

**Authors:** Mehmet Mustafa Yılmaz, Osman Karaarslan, Macit Kalçık

**Affiliations:** ^1^ Department of Cardiology Hitit University Erol Olçok Education and Research Hospital Corum Turkey; ^2^ Department of Cardiology, Faculty of Medicine Hitit University Corum Turkey

To the Editor,

We read with interest the recent study by Happ et al. (2026) evaluating physician perspectives regarding the preferred initial diagnostic strategy in older patients presenting with unexplained syncope without structural or electrical heart disease. The authors provide valuable insight into contemporary practice patterns and highlight the absence of consensus between implantable cardiac monitoring and tilt table testing as the initial diagnostic modality. However, several methodological and interpretive considerations warrant further discussion before these preference‐based findings are translated into clinical decision‐making frameworks.

One important limitation relates to the interpretation of physician preference as a surrogate for optimal diagnostic sequencing. Surveys primarily reflect perceived diagnostic utility rather than objective effectiveness, and clinical decision‐making in syncope evaluation is expected to be guided by structured diagnostic pathways rather than subjective preference alone. Current international guidelines emphasize risk stratification and clinical context when selecting monitoring strategies, rather than endorsing a uniform ICM‐first or TTT‐first approach (Shen et al. 2017).

Another issue concerns the representativeness of the study population. The majority of respondents were electrophysiologists, which may have introduced specialty‐related bias favoring rhythm‐monitoring strategies such as implantable cardiac monitoring. Previous registry data demonstrate that ICM use increases diagnostic yield in unexplained syncope, but these benefits are particularly evident in carefully selected patient populations rather than universally across all older adults with nondiagnostic initial evaluation (Edvardsson et al. 2011). Therefore, extrapolating these survey findings to broader clinical practice should be performed cautiously.

The observed discrepancy between physician concerns about missed diagnoses with tilt testing and their stated prioritization of rapid diagnostic yield also deserves closer consideration. Tilt testing has been shown to provide an early diagnosis in a substantial proportion of patients with reflex syncope and remains a guideline‐supported tool in patients without clear arrhythmic suspicion. Meta‐analytic evidence suggests that structured tilt testing protocols may achieve meaningful diagnostic rates within a short time frame, which contrasts with the delayed diagnostic timeline typically associated with prolonged monitoring strategies (Forleo et al. 2013).

Finally, the regional variability in preferred diagnostic strategies reported in the study likely reflects differences in infrastructure availability and training patterns rather than intrinsic differences in test performance. Previous observational studies have demonstrated that implantable loop recorders improve diagnostic certainty in selected patients but do not replace structured clinical evaluation or reflex syncope assessment pathways. Accordingly, diagnostic sequencing should remain individualized and evidence‐based rather than preference‐driven (Brignole et al. 2018).

In conclusion, the study by Happ et al. provides important insight into physician attitudes toward diagnostic sequencing in unexplained syncope. Nevertheless, survey‐derived preferences should be interpreted cautiously and should not substitute for guideline‐directed and patient‐specific diagnostic strategies when determining the optimal first‐line investigation.

## Author Contributions

All of the authors contributed to the planning, writing, and revision.

## Funding

The authors have nothing to report.

## Conflicts of Interest

The authors declare no conflicts of interest.

## Data Availability

Data sharing not applicable to this article as no datasets were generated or analysed during the current study.
